# Modulation of CD44 Activity by A6-Peptide

**DOI:** 10.3389/fimmu.2015.00135

**Published:** 2015-03-30

**Authors:** Malcolm Finlayson

**Affiliations:** ^1^Ångstrom Pharmaceuticals Inc., Solana Beach, CA, USA

**Keywords:** A6, CD44, HA, CLL, metastasis, recurrence, resistant, ocular

## Abstract

Hyaluronan (HA) is a non-sulfated glycosaminoglycan distributed throughout the extracellular matrix that plays a major role in cell adhesion, migration, and proliferation. CD44, a multifunctional cell surface glycoprotein, is a receptor for HA. In addition, CD44 is known to interact with other receptors and ligands, and to mediate a number of cellular functions as well as disease progression. Studies have shown that binding of HA to CD44 in cancer cells activates survival pathways resulting in cancer cell survival. This effect can be blocked by anti-CD44 monoclonal antibodies. A6 is a capped, eight l-amino acid peptide (Ac-KPSSPPEE-NH_2_) derived from the biologically active connecting peptide domain of the serine protease, human urokinase plasminogen activator (uPA). A6 neither binds to the uPA receptor (uPAR) nor interferes with uPA/uPAR binding. A6 binds to CD44 resulting in the inhibition of migration, invasion, and metastasis of tumor cells, and the modulation of CD44-mediated cell signaling. A6 has been shown to have no dose-limiting toxicity in animal studies. A6 has demonstrated efficacy and an excellent safety profile in Phase 1a, 1b, and 2 clinical trials. In animal models, A6 has also exhibited promising results for the treatment of diabetic retinopathy and wet age-related macular degeneration through the reduction of retinal vascular permeability and inhibition of choroidal neovascularization, respectively. Recently, A6 has been shown to be directly cytotoxic for B-lymphocytes obtained from patients with chronic lymphocytic leukemia expressing the kinase, ZAP-70. This review will discuss the activity of A6, A6 modulation of HA and CD44, and a novel strategy for therapeutic intervention in disease.

## Introduction

Mortality due to cancer is generally the result of metastasis of the primary tumor. Recurrence at distant sites following first-line therapy continues to be a major challenge. As a result, drugs that inhibit the metastatic process are of great interest. Metastasis and recurrence have been linked to a subpopulation of highly invasive tumorigenic cells that are characterized by the expression of CD44. A6 has been shown to bind to CD44 and to exhibit anti-metastatic properties. Thus, A6 may serve as a therapeutic alternative for the treatment and prevention of metastatic disease.

### A6 background

A6 is a capped eight l-amino acid peptide (Ac-KPSSPPEE-NH_2_) derived from amino acid residues 136–143 of the connecting peptide domain of human urokinase plasminogen activator (uPA).

**Figure 1:**
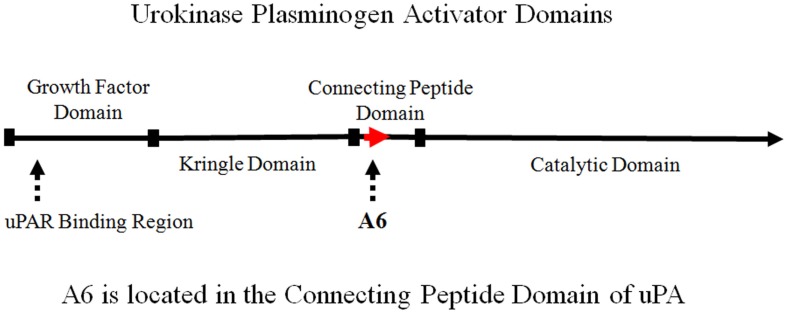
**Schematic representation of urokinase plasminogen activator (uPA) domains illustrating the location of A6**.

The connecting peptide domain is located between the N-terminal growth factor domain and the C-terminal catalytic domain of uPA (Figure 1). The N-terminal growth factor domain of uPA binds to the uPA receptor (uPAR) to initiate the uPA/uPAR cascade, which is catalyzed by the C-terminal serine protease that activates plasminogen to plasmin. Numerous studies have shown that the binding of uPA to uPAR, initiates a cascade of events leading to proteolysis, degradation of the extracellular matrix (ECM), cell migration, cell invasion, metastasis, and angiogenesis (1–4). Further, the uPA system has been shown to play an important role in the growth and spread of solid tumors. Levels of uPA and uPAR correlate with clinical outcome in a variety of malignancies. Specifically, the upregulation of the uPA system is associated with poor prognosis, and inhibition of the uPA system has been shown to block critical processes (e.g., cell migration, invasion, and angiogenesis) required for a broad range of proliferative diseases. This provides the rationale for development of inhibitors of this pathway (5–9).

The binding of uPA to uPAR has been localized to the N-terminal growth factor domain. Although the growth factor domain is known to initiate biological activity, the connecting peptide domain of uPA has also been shown to have biological activity independent of the growth factor domain (10, 11). uPA has been shown to bind to HEK 293 cells (transformed with uPAR) to stimulate migration (10). The binding of uPA peptide fragments composed of the growth factor domain and connecting peptide domain were evaluated to characterize domain-specific activity on the cell surface (10). Both the growth factor and connecting peptide domains were capable of stimulating cell migration independently. Furthermore, the binding of these peptide fragments to HEK 293 cells was inhibited by increasing concentrations of the uPA molecule. However, the connecting peptide domain itself did not inhibit growth factor domain binding, and growth factor domain did not inhibit connecting peptide domain binding. This indicated the presence of distinct binding sites for both the growth factor domain and connecting peptide domain, as well as a uPAR-independent signaling pathway (10). It was postulated that the connecting peptide domain of uPA was functioning through interaction with a cell surface integrin receptor (10, 11).

**Figure 2:**
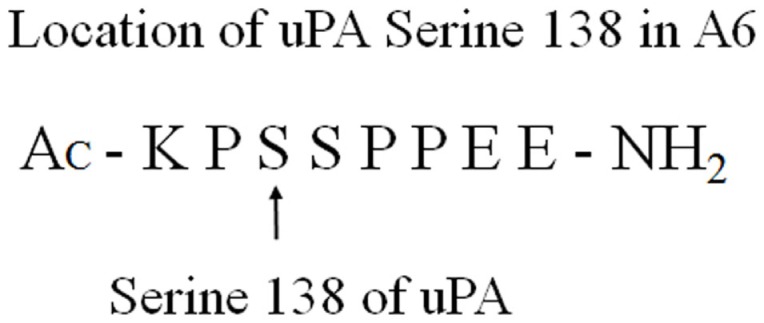
**Serine 138 of uPA is included in the A6-peptide sequence**.

The biological importance of the connecting peptide domain (amino acid residues 132–158) has been further demonstrated by the phosphorylation or substitution of serine 138, which results in the inhibition of uPA-induced cell migration without changing uPA binding to uPAR (12, 13). A6 comprises uPA amino acids 136–143. The serine 138 residue of uPA is included in the A6 sequence (Figure 2).

Phosphorylation or substitution of uPA serine 138 does not change the growth factor domain-mediated binding of uPA to uPAR, yet inhibits migration, which suggests that the connecting peptide domain region is important for regulatory function. This indicates that simultaneous concurrent interaction of uPA domains, with distinct surface receptors, is required for uPAR-dependent cell migration. This provided additional evidence to support the investigation of the A6-peptide.

The biological activity localized within the uPA connecting peptide domain prompted additional study. It was postulated that proteolysis by plasmin and matrix metalloproteinases (MMPs) such as MMP3 and MMP7 could excise a fragment from amino acids 136–143, corresponding to A6. Although there is no evidence that this proteolytic processing occurs, several peptides comprising various fragments of the connecting peptide domain sequence were synthesized. The A6 sequence was found to have activity, which led to the preclinical and clinical investigation of A6.

**Figure 3:**
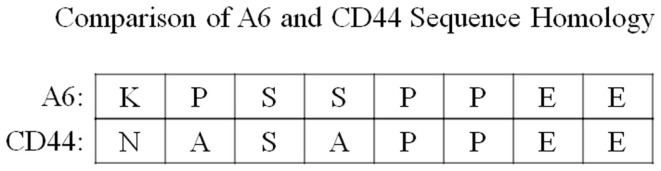
**A comparison of the sequence homology between A6 and amino acid residues 120–127 of the CD44 link domain**.

Interestingly, A6 shares sequence homology with a portion of the link domain of CD44 (CD44 amino acid residues 120-NASAPPEE-127) (14) (Figure 3). The CD44 gene is encoded by 20 exons in the mouse and 19 exons in humans: 5 constant exons are expressed at the 5′ end, and 10 variant exons (mouse) or 9 variant exons (human) may be alternatively spliced within CD44 at an insertion site after the fifth constitutive exon, followed by the remaining constant exons at the 3′ end (15, 16). The standard isoform of CD44 (CD44s) is the smallest isoform containing no variant exons, and the largest is CD44v1–10, which contains all of the variant exons. Although a CD44 isoform specificity has not been determined for A6, it is important to note that the region of A6 sequence homology in CD44, through which A6 may act, is conserved in all CD44 isoforms as it is located within the first five non-variable exons of the molecule (15). This homology is important because the sequence straddles the CD44 splice junction of exons 3 and 4, includes a potential glycosylation site, and is involved with hyaluronan (HA) binding (14, 15, 17–21). The evaluation of A6 activity using alanine scanning mutagenesis demonstrated that there is a degree of substitution that can be accommodated without a substantial loss of A6 activity, and analysis of the CD44 homologous peptide sequence revealed activity similar to that of A6 (22).

## A6 and Metastatic Disease

### Preclinical studies

Preclinical studies have shown that A6 has anti-migratory, anti-invasive, and anti-metastatic properties. A6 has been shown to inhibit migration and invasion of breast, lung, glioma, ovarian, and prostate cancer cell lines *in vitro* in a dose-dependent manner (23–27), and to inhibit the growth and metastasis of breast, melanoma, glioma, lung, and prostate cancer cells in xenograft models *in vivo* (14, 24–27). Interestingly, the combination of A6 with tamoxifen resulted in an inhibition of breast tumor cell growth greater than with either A6 or tamoxifen alone (24). A similar result was observed in glioma xenograft studies where the combination of A6 with cisplatin also inhibited tumor cell growth greater than with either A6 or cisplatin alone (26). These results are important because of the relationship between CD44 and chemoresistance.

### *In vitro* studies

Boyden chamber analyses demonstrated that A6 inhibited chemotaxis in a variety of human breast and ovarian cancer cell lines in a concentration-dependent manner (14). The IC_50_ for the inhibition of chemotaxis of responsive cell lines was 10–100 nmol/L suggesting physiological relevance (14). Furthermore, A6 inhibition of chemotaxis was shown to correlate with the expression of CD44. This was demonstrated by flow cytometric analysis with four different anti-CD44 antibodies and five different human ovarian cancer cell lines. A6 produced more than 85% inhibition of migration in CD44-positive SKOV3 cells when compared to untreated control (14). Notably, A6 had no effect on the migration of CD44-negative A2780 cells. A6 was also shown to interfere with the binding of only one (DF1485) of the four anti-CD44 antibodies tested (14). A6 did not interfere with the binding of the anti-CD44 antibody, IM7, which blocks HA binding to CD44. These findings suggest that A6 does not produce a global non-specific change in CD44, but instead produces a subtle change to a specific epitope.

Because A6 inhibited migration of SKOV3 cells, this study also examined the direct interaction of A6 with CD44 (14). Human ovarian SKOV3 cells were bound and cross-linked to A6. Immunoprecipitation and immunoblotting of lysate preparations of cross-linked cells revealed that A6 was binding to CD44. To determine if this binding influenced CD44-mediated activity, and to determine if a functional relationship existed between A6 and CD44, intracellular signaling studies were conducted. A6 was shown to modulate FAK phosphorylation in CD44-positive SKOV3 cells, but not in CD44-negative A2780 cells. The study further demonstrated that the A6 modulation of FAK phosphorylation in SKOV3 cells was blocked by HA. These results show that a functional relationship exists between A6 and CD44 binding and CD44-mediated intracellular signaling (14).

### *In vivo* studies

#### Mammary

The effects of A6 in mammary tumor and metastasis models have been investigated. Studies with BALB/c (nu/nu) mice implanted with MDA-MB-231 human mammary carcinoma xenografts demonstrated that A6 inhibited tumor growth by 90% compared to control (23). An inhibition of metastasis was also noted. Additionally, the effect of A6 in Fisher rats inoculated with Mat B-III syngeneic mammary carcinoma cells was evaluated. A6 treatment inhibited tumor growth by 55% and markedly suppressed lymph node metastasis (23). Furthermore, the combination of A6 with tamoxifen in Fisher rats with Mat B-III syngeneic mammary carcinoma resulted in a 75% inhibition of tumor growth (24).

#### Prostate

A model of prostate cancer was used to evaluate the anti-metastatic effect of A6 in mice. Metastases to lymph nodes were measured following the orthotopic injection of human PC-3M-LN4 prostate cancer cells into the prostates of BALB/c (nu/nu) mice. The percentage of mice with lymph node metastases was reduced from more than 70% in the control group to as low as 22% in A6-treated animals (27). Additionally, A6 treatment significantly reduced lymph node volume by as much as 70%.

#### Glioblastoma

In animal models of glioblastoma, U87MG human glioma cells were implanted subcutaneously or intracranially in BALB/c (nu/nu) mice and the animals were divided into different treatment groups. A6 treatment suppressed subcutaneous U87MG tumor growth by 48% and prolonged the time to progression (TTP) following discontinuation of A6 treatment (26). In this study, the effects of cisplatin were also examined. Cisplatin treatment reduced tumor growth by 53%. Interestingly, the combination of A6 and cisplatin resulted in a 92% inhibition of subcutaneous tumor growth. This result was consistent with a U87MG intracranial xenograft study in which mice receiving a combination of A6 and cisplatin exhibited a significantly greater inhibition of tumor growth (98%) when compared to either A6 (44%) or cisplatin (82%). In this study, the combination therapy also significantly increased survival time over that for either drug alone. This was consistent with subcutaneous xenograft results.

#### Melanoma

The well-characterized B16-F10 lung metastatic model was employed to determine the ability of A6 to inhibit the colonization of secondary tissues by circulating cancer cells (14). B16-F10 melanoma cells were evaluated by flow cytometric analysis and were shown to express CD44. The IC_50_ for A6 inhibition of chemotaxis in B16-F10 cells was 29 nmol/L, indicative of a responsive cell line. Melanoma cells were injected into the tail veins of C57BL/6 mice to simulate a burden of metastasizing cells and the lungs were then evaluated for lesions at day 11. Treatment with A6 reduced the number of lung metastases to 50% of control. Taken with previous results, this is important because it demonstrates that A6 not only inhibits the initial steps of the metastatic process (e.g., migration and invasion) but also inhibits the formation of secondary lesions after tumor cells enter the circulation.

#### Leukemia

A6 has also been evaluated for activity in hematological malignancies. Chronic lymphocytic leukemia (CLL) is characterized by the accumulation of mature monoclonal B cells in the blood and secondary tissues. CD44 is highly expressed in CLL cells and mediates the interaction between CLL cells and the microenvironment. CLL cells receive survival signals from the microenvironment, and one of these pathways is mediated by CD44. Binding of HA to CD44 has been shown to activate PI3K/AKT and MAPK/ERK-mediated survival pathways, and to induce expression of the anti-apoptotic protein Mcl-1, which promotes CLL cell survival (28). It has been shown that this effect can be blocked by an inhibitor of Mcl-1, or by anti-CD44 monoclonal antibodies, leading to apoptosis *in vitro* (29, 30).

Recent studies (31, 32) with human CLL B-cell lymphocytes have shown that A6 down modulates the expression of CD44 and ZAP-70 (a marker for an aggressive form of CLL), and inhibits B-cell receptor (BCR) signaling, resulting in a direct, dose-dependent, cytotoxicity *in vitro*. To evaluate the effects of A6 *in vivo*, an established CLL xenograft model was employed. ZAP-70^pos^ B-cell lymphocytes isolated from individual patients were injected into immune-deficient mice treated with A6 or vehicle control. A6 treatment resulted in up to 90% reduction in CLL burden (31, 32). Previously, A6 had not demonstrated cytotoxicity in solid tumor models of glioma, breast, and ovarian cancer (14, 22, 23, 25). However, in these CLL studies, A6 was shown to be directly cytotoxic for CLL B-cell lymphocytes. A6 is currently being evaluated for the treatment of CLL.

### Clinical studies

Several clinical studies have been conducted to evaluate the safety and efficacy of A6. These include safety studies in healthy volunteers as well as studies in patients with varying stages of metastatic disease.

#### Normal volunteers

A6 was administered to normal volunteers in a Phase 1a, double-blind, placebo-controlled, parallel-group clinical trial (33). Results showed that there were no systemic drug-related adverse events. No significant alterations in physical examinations, vital signs, electrocardiograms, or clinical laboratory testing, including coagulation parameters such as PT, PTT, fibrinogen, and thrombin time, were noted. Pharmacokinetic data in normal volunteers at the 150 and 300 mg/day single dose levels showed a *t*_1/2_ of 1.8–2.0 h at both dose levels. Furthermore, no cumulative increase in concentration over time was detected. Following A6 subcutaneous administration twice daily for 6 days, no anti-A6 antibody production was detected at day 14 (33).

#### Advanced gynecologic cancer

A Phase 1b trial was conducted in women with advanced gynecologic cancer (34). Greater than 40% of patients dosed continuously with A6 experienced disease stabilization. The study used a sequential dose-escalation design, with the lowest-dose group (four patients) receiving A6 for cycles of 14 days “on” followed by 14 days “off,” a regimen not expected to produce any therapeutic effect. Twelve patients with advanced gynecologic malignancies that had failed standard therapy were treated with daily, uninterrupted A6. In this population, in which disease progression is expected, five patients (four of whom had ovarian or primary peritoneal carcinoma) achieved stable tumor measurements for at least 4 months, and one for greater than 12 months. Patients continued treatment until disease progression or unacceptable toxicity. Response was evaluated as defined by RECIST and the Gynecologic Cancer Intergroup (GCIG) CA-125 response criteria. A Kaplan–Meier retrospective analysis demonstrated that patients treated with daily A6 showed a delayed time to tumor progression relative to an effective control group (whose treatment was intermittent and, therefore, not expected to have beneficial effect) providing evidence of antineoplastic activity. Continuous treatment with A6 resulted in an increased TTP with a median TTP of 78 days (95% CI 57, 365) compared to 44 days (95% CI 4, 62) in patients who received the intermittent therapy (log-rank *p*-value = 0.02). The safety outcome in this Phase 1b gynecologic cancer trial was excellent and showed no specific toxicity profile.

#### Asymptomatic progression of ovarian cancer

A randomized, double-blind, placebo-controlled Phase 2 clinical trial evaluating A6 in women with asymptomatic CA-125 progression of ovarian cancer (“marker-only relapse” or MOR) was conducted (35). Patients were in clinical remission after first-line chemotherapy with no evidence of disease following physical examination or imaging analysis, but had two consecutive, above-normal, increases of CA-125 (a biomarker for recurrence/poor prognosis). Because patients were clinically asymptomatic at the time of entry, the study was able to be placebo-controlled. The primary endpoints were time to clinical progression of disease and safety of A6. The secondary endpoints included changes in serum CA-125. This study enrolled 24 patients: 12 were randomized to daily self-administration of A6 at two doses, and 12 to matching placebo injections. Both groups were followed for up to 9 months. Although there were no complete responses, 36% of patients achieved stable disease. A6 treatment was not associated with CA-125 response. Results from a Kaplan–Meier analysis of progression-free survival (PFS) showed that treatment with A6 significantly prolonged TTP. Despite the small patient sample size, A6 therapy was associated with a statistically significant increase in PFS (log-rank *p*-value = 0.01) with a median PFS of 100 days (95% CI 64, 168) compared to 49 days (95% CI 29, 67) in patients who received the placebo. Furthermore, the safety profile of A6 was comparable to that of control (placebo) treatment.

#### Persistent or recurrent ovarian cancer

A Phase 2 trial was conducted in patients with persistent or recurrent epithelial ovarian, fallopian tube, or primary peritoneal carcinoma (36) to evaluate A6 in a patient population with a disease burden greater than that presented in the previously described MOR trial. Patients had received one prior platinum-based chemotherapeutic regimen and were allowed to have received one additional cytotoxic regimen for the management of recurrent or persistent disease. Patients received a 150 mg twice daily subcutaneous dose of A6 and continued on treatment until disease progression or unacceptable toxicity. Response criteria were as defined by RECIST. Primary measures of clinical efficacy were objective tumor response and PFS at 6 months compared to a historical Gynecologic Oncology Group (GOG) dataset based on a similar population of patients. Of the 31 eligible patients evaluated, no responses were observed; 6.5% were progression free for at least 6 months; and 36% of evaluable patients achieved stable disease. A6 was well tolerated but had minimal activity in patients with persistent or recurrent epithelial ovarian, fallopian tube, or primary peritoneal carcinoma under the conditions of this trial. Considering the relationship of A6 to CD44 and the relationship of CD44 to resistant and recurrent disease, it would be of interest to follow this study with a combination trial comparing standard-of-care to standard-of-care plus A6 in this difficult population.

## A6 and Ocular Disease

A6 has been evaluated for the treatment of ocular disease (37). The focus of this application has been wet age-related macular degeneration (AMD) and diabetic retinopathy, which are characterized by neovascularization and vascular permeability. Since angiogenesis is known to involve HA and to be mediated by CD44 (38–40), A6 has been investigated for use as a therapeutic for these conditions. Angiogenesis is a multistage process involving cell migration and ECM remodeling, including the loss of cellular structure and function followed by invasion. Similar cellular changes are also observed early in the metastatic process. These cellular changes can be more accurately described in terms of an epithelial–mesenchymal transition (EMT). EMT is a process by which epithelial cells acquire mesenchymal-like properties, with reduced intercellular adhesion and increased motility, critical to many developmental, homeostatic, and pathological processes. The EMT process is a continuum leading to enhanced cell migration and invasion. Preceding migration, there is a loss of cadherin and epithelial adhesion, followed by disruption of the basement membrane and degradation of the ECM by MMPs (41–45). A6 has been shown to inhibit this process.

### Wet age-related macular degeneration

Several *in vivo* studies have been conducted to evaluate the efficacy of A6 for the treatment of wet AMD. In the mouse model of laser-induced choroidal neovascularization (CNV), treatment with A6 resulted in a 95% inhibition of new vessel formation compared to the non-treated control group (46). Results employing a rat model of laser-induced CNV showed that subcutaneous injections of A6 produced a 70% reduction in CNV compared to non-treated controls (47). Finally, results from a primate model of laser-induced CNV demonstrated that intravitreal administration of A6 resulted in a 71% reduction in CNV relative to control (48). These studies demonstrate that A6 may be a promising candidate for the treatment of wet AMD.

### Diabetic retinopathy

Research involving the use of A6 for treatment of diabetic retinopathy demonstrated that A6 treatment prevents the loss of vascular endothelial (VE)-cadherin and inhibits the increase in microvascular permeability in the retina of diabetic Brown Norway rats induced with streptozotocin (49). In the same study, similar results were observed using bovine retinal microvascular endothelial cells and showed that VE-cadherin degradation was associated with increased vascular permeability and the secretion and activation of MMP-2 and MMP-9. Treatment with A6 was shown to inhibit MMP-dependent VE-cadherin degradation and the loss of permeability. In addition, A6 prevented the secretion and activation of MMP-2 and MMP-9 (49). HA has also been shown to increase MMP-2 and MMP-9 expression in cell culture (50) and to promote CD44-EGFR interaction leading to MMP-2 secretion and enhanced cell motility (51). The ability of A6 to inhibit MMP activation may have important implications for the metastatic process.

The role of hepatocyte growth factor (HGF) in angiogenesis (52, 53) as well as the elevated intravitreous concentrations of HGF in diabetic patients has been described (54, 55). The effect of A6 on HGF and its receptor, c-Met, in retinal angiogenesis has been examined (56). This study demonstrated that HGF was upregulated in the retinas of mice following hypoxia-induced retinal neovascularization. Furthermore, HGF was shown to stimulate retinal microvascular endothelial cell invasion *in vitro*, which is consistent with the angiogenic process. HGF-induced retinal endothelial cell invasion was reduced to control levels following treatment with A6 (56). Since CD44 functions as a co-receptor with c-Met, these results suggest a possible mechanistic pathway for A6.

## Discussion

The metastatic process involves migration and invasion of tumor cells from the local microenvironment, intravasation into the blood or lymph circulation, extravasation from circulation back into tissue, followed by metastatic colonization and growth or dormancy (57). Metastasis and recurrence have been linked to a subpopulation of highly invasive tumorigenic cells, which have been shown to be resistant to chemotherapeutics. These tumorigenic cells are characterized by the expression of CD44, a multifunctional receptor involved in cell signaling, adhesion, migration, and proliferation. CD44 functions as a receptor, as a co-receptor (e.g., c-Met and EGFR), and as a platform for MMPs to enable many biological processes (58, 59). In addition, CD44 is known to mediate invasion and metastasis (60).

Chemotherapeutic resistance has been linked to a number of CD44 pathways including MDR1-dependent efflux of chemotherapeutics (61–65). This resistance results in expansion of invasive cells following first-line chemotherapy, which leads to recurrence. Studies have shown that targeting CD44 or related signaling pathways, using RNAi strategies (61, 62, 66, 67) or with anti-CD44 antibodies (68), will suppress tumor growth and relapse, and increase sensitivity of these cells to chemotherapeutics. In animal xenograft models, A6 has been found to enhance the activities of both tamoxifen to inhibit the growth of breast tumor cell growth (24) and cisplatin to inhibit the growth of glioma cells (26). This supports the concept that targeting CD44 may render tumor cells more sensitive to therapeutic agents.

A6 has been shown to bind to CD44 and to modulate CD44-mediated activity. A6 demonstrates anti-metastatic properties by inhibiting migration and invasion, which are early steps in the metastatic process. The mechanism by which A6 acts may involve inhibition of EMT, as observed in studies of ocular disease where A6 inhibited MMP activation and cadherin degradation (49). Rationale for this consideration is supported by evidence that HA is implicated in MMP activation (50, 51). A6 also acts later in the metastatic process to inhibit the formation of lesions resulting from the direct injection of cancer cells into the blood stream (14). This would indicate that A6 inhibits steps involving extravasation and/or metastatic colonization. This is important when considering recurrence following adjuvant therapy and the possibility of proliferation of dormant micrometastases long term.

Dormancy and micrometastases present a therapeutic challenge (69, 70). That subclinical micrometastases may be present long-term was demonstrated in a study involving 36 breast cancer patients found to be disease free from 8 to 22 years post-resection (71). This study demonstrated that in one-third of these patients, with no evidence of disease, viable circulating tumor cells (CTCs) could be isolated. The CTCs were determined to be non-proliferative with a short half-life, but were found when repeated samples were taken up to 2 years after the patients entered the study. This suggested that CTCs were being continuously released from subclinical micrometastases. Long-term or maintenance therapy targeting recurrence is not practical when considering many cytotoxic agents. However, due to its superior safety profile (no immunogenicity, no dose-limiting toxicities, no serious side-effects), long-term or maintenance therapy with A6 may be an option. The use of A6 in this manner could introduce a new paradigm to cancer treatment.

As previously noted, A6 shares sequence homology with the link module of CD44. The link module of CD44 has been shown to be critical to HA binding and cell migration. When the CD44 link module was substituted with a homologous region of higher HA affinity (TSG-6), cells expressing this chimera bound HA, but failed to migrate and were described as tethered (20). A6 was shown to increase the binding of CD44-expressing SKOV3 cells to HA-coated plates (14). This effect was blocked with the anti-CD44 antibody, IM7. However, neither A6 nor IM7 had any effect on the binding of CD44-non-expressing A2780 cells to HA-coated plates. These results suggest that increasing adhesion may play a role in the anti-metastatic activity of the A6-peptide, and again illustrate correlation of A6 activity with CD44 expression. The study further demonstrated that A6 perturbed the binding of the anti-CD44 antibody, DF1485, to CD44-expressing SKOV3 cells. This was reported to be a partial inhibition, which did not result from a competition involving either A6 or CD44. Furthermore, the DF1485 antibody did not recognize A6 or inhibit the binding of an anti-A6 antibody to A6. It was postulated that A6 induces conformational changes in CD44, resulting in either a lowered affinity of the epitope for DF1485, or preventing DF1485 from binding (14). Regardless of the mechanism, the binding of A6 to CD44 results in a modulation of CD44-mediated intracellular signaling (14). This establishes a functional relationship between A6 and CD44 in CD44-expressing cells. This functional relationship was demonstrated by monitoring FAK phosphorylation in the presence and absence of A6 and HA in CD44-expressing and -non-expressing cell lines. Although HA is reported to have a rheostatic effect on CD44, with high molecular weight HA inhibiting tumor progression, and low molecular weight HA stimulating tumor progression (72–74), the extent to which A6 may differentiate these activities has yet to be studied.

The precise mechanism of A6 has not been defined, but compelling evidence from studies on metastatic and ocular disease supports action through a CD44-mediated pathway. Whether this is by direct action on CD44 or by modulating CD44 co-receptor activity remains unclear. A6 binding to CD44 and the effects of A6 on chemotaxis and intracellular signaling have been demonstrated in the absence of HA. This indicates that A6 has a primary effect, through CD44, that is independent of HA. However, because A6 increases adhesion of CD44-expressing cells to HA, it also suggests that A6 may interact with HA secondarily to A6 binding to CD44. The CD44 ligand-binding region that shares homology with A6 is likely to be critical to the mechanism by which A6 modulates the activity of CD44. A6 may simulate the CD44 sequence and trigger a homotypic interaction resulting in modification of CD44 activity by inducing a conformational change in CD44, or CD44 dimerization, or both. As mentioned, the perturbation of DF1485 binding by A6 suggests that A6 may induce conformational changes in the receptor. Alternatively, A6 homology may simulate the CD44 sequence permitting it to influence a CD44-binding partner/co-receptor resulting in modulation of CD44-mediated activity. This is supported by A6 inhibitory activity on HGF and MMPs observed in ocular diseases. Finally, although A6 binds to CD44, the possibility cannot be excluded that A6 interacts with a protein independent of CD44 that initiates secondary modulation of CD44 activity.

CD44 is a complex multifunctional receptor modulating a variety of cellular processes. Although the mechanistic process is not completely defined, the studies described have demonstrated that A6 inhibits the metastatic process in a CD44-dependent manner. Because CD44 is associated with a chemoresistant phenotype, which is countered by inhibition of CD44 signaling, A6 is a candidate for inhibition of CD44-mediated resistance. In this case, A6 would be used in combination with a cytotoxic chemotherapeutic agent to inhibit metastases and to render resistant cells sensitive to chemotherapy. Certainly, the results from preclinical A6 combination studies in animals support this approach. Furthermore, due to the positive safety profile documented for A6, there would be a reduced likelihood of compounding toxicity. As such, A6 may potentially be combined with almost any chemotherapeutic. This safety profile also invites the use of A6 for longer-term maintenance therapy to prevent recurrence stemming from micrometastases surviving first-line standard-of-care treatment. A6 has demonstrated activity against CD44-expressing tumor cells and CLL cells, and is a candidate for the treatment of malignant disease and hematological malignancy. A6 has demonstrated clinical safety and efficacy, and by targeting CD44-resistant cells to prevent metastases and recurrence, has the possibility of creating a new paradigm for cancer treatment.

## Conflict of Interest Statement

The author has a financial relationship with Angstrom Pharmaceuticals, Inc.
